# Antitumor Effect of Curcumin, D6 Turmeric, and Hydrochloride Mitoxantrone on Canine and Human Urothelial Cancer Cells

**DOI:** 10.3390/ani15111589

**Published:** 2025-05-29

**Authors:** Thayná Oliveira da Silva, Luís Gustavo Ramos de Moraes Calheiros, Felipe Barbosa, Fernanda Bueno Morrone, Liliana Rockenbach, Patrícia de Faria Lainetti, Antonio Fernando Leis Filho, Márcio de Carvalho, Carlos Eduardo Fonseca-Alves, Renée Laufer Amorim

**Affiliations:** 1 Department of Veterinary Clinic, São Paulo State University-UNESP, Botucatu 18618-681, SP, Brazil; thayna.o.silva@unesp.br (T.O.d.S.);; 2 Laboratory of Applied Pharmacology, School of Health and Life Sciences, Pontifical Catholic University of Rio Grande do Sul, Porto Alegre 90619-900, RS, Brazil; 3 Institute of Health Sciences, Paulista University–UNIP, Bauru 18618-681, SP, Brazil

**Keywords:** apoptosis, cytotoxicity, gene expression, cell migration, epithelial–mesenchymal transition

## Abstract

Bladder urothelial carcinoma (UC) is an aggressive malignancy in both humans and dogs, with limited treatment options. Owing to their biological and environmental similarities with humans, dogs serve as a valuable model for UC research. Standard treatments, including surgery, chemotherapy, and anti-inflammatory agents, have shown limited efficacy. Curcumin, a bioactive compound derived from turmeric, has demonstrated anticancer properties, but its potential in canine UC remains poorly understood. In this study, we evaluated the effects of curcumin, D6 turmeric, and mitoxantrone hydrochloride on canine and human UC cell lines. Cell viability was assessed via the MTT assay, apoptosis via flow cytometry, and gene expression (*β-catenin*, *β1-integrin*, *CDH1*, *MMP-2*, *MMP-9*, and *TIMP-2*) via quantitative PCR. Migration capacity was analyzed using a Transwell assay. Curcumin and D6 turmeric reduced cell viability and migration, while mitoxantrone hydrochloride exhibited strong cytotoxicity, especially in canine cells. Curcumin also induced apoptosis and modulated genes involved in epithelial–mesenchymal transition and invasion. The interindividual differences in response suggest underlying genetic variability and highlight the need for personalized therapeutic approaches. These findings suggest that curcumin and D6 turmeric hold promise as complementary therapies for canine UC, justifying further in vivo investigations.

## Simple Summary

Bladder cancer is a challenging disease in both humans and dogs, and current treatments such as surgery and chemotherapy often yield limited success. Because dogs naturally develop this cancer and share many clinical and biological features with humans, they are valuable models for investigating new therapies. In this study, we tested curcumin—a natural compound derived from turmeric—alongside a chemotherapeutic agent on bladder cancer cell lines from both species. Our results demonstrated that curcumin decreased cancer cell viability, reduced migration, and increased apoptosis. The chemotherapeutic drug showed pronounced cytotoxic effects in canine cells. These findings support the potential use of curcumin as an adjuvant to conventional therapies, possibly improving outcomes in dogs with bladder cancer. Further research is needed to better understand curcumin’s mechanisms and safety profile in vivo. This study lays the groundwork for the future development of novel and less toxic therapeutic strategies that could benefit both veterinary and human medicine.

## 1. Introduction

Cancer remains one of the most life-threatening diseases and a significant global public health concern. In 2020, bladder cancer was identified as the 10th most common malignancy worldwide, with approximately 573,278 new cases—441,000 in men and 132,000 in women [1]. In the United States alone, it was estimated that 82,290 new cases and 16,710 deaths would occur in 2023 [2]. Among bladder cancers, urothelial carcinoma (UC), also known as transitional cell carcinoma, is the predominant histological type. It may arise in the renal pelvis, ureter, bladder, or urethra. UC is classified as either non-invasive or invasive, with approximately 25% of patients developing muscle-invasive disease that may lead to metastasis [1].

In dogs, UC accounts for approximately 2% of all urinary tract neoplasms and represents over 90% of bladder malignancies. Approximately 10,000 new canine cases are reported worldwide annually [3,4,5]. Clinical signs, such as hematuria, dysuria, and urinary obstruction, are non-specific and overlap with those of other lower urinary tract disorders, often resulting in delayed diagnosis [6]. Most canine UCs are high-grade invasive tumors, typically located in the trigone region, and may involve the urethra or prostate in males [4].

Canine and human UC share notable similarities in histopathological features, tumor behavior, therapeutic responses, and overall prognosis. Furthermore, dogs and their owners are exposed to similar environmental carcinogens [7,8]. Interestingly, canine UC tends to be more aggressive, making the species a valuable natural model for studying advanced or invasive forms of the disease in humans.

Treating UC in veterinary medicine poses several challenges, including tumor location, disease progression, and frequent late-stage diagnosis. Treatment options include surgery, chemotherapy, cyclooxygenase-2 (COX-2) inhibitors, radiotherapy, or multimodal approaches [9,10]. However, surgical intervention is often not feasible due to tumor localization in the trigone, which increases the risk of urinary incontinence and may compromise the animal’s quality of life [11].

Several chemotherapeutic agents have been employed to manage canine UC, such as cisplatin, carboplatin, mitoxantrone, actinomycin D, and doxorubicin [12]. Although chemotherapy is not curative, it remains a cornerstone for disease control or remission. Median survival times reported for systemic chemotherapy combined with piroxicam are approximately 181 days, compared to 291 days for mitoxantrone combined with piroxicam [4].

The therapeutic use of plant-derived compounds has a long history and is increasingly recognized for its potential in oncology [13]. *Curcuma longa* L., a member of the Zingiberaceae family native to India, contains curcumin as its most abundant and active curcuminoid, accounting for approximately 77% of its composition. Other curcuminoids include desmethoxycurcumin and bisdesmethoxycurcumin [14]. Curcumin is known for its diverse biological activities, including antioxidant, anti-inflammatory, antibacterial, antifungal, antitumor, antiviral, healing, hypoglycemic, neuroprotective, antiparasitic, and immunomodulatory effects [14,15].

The antitumor potential of curcumin has been demonstrated in several human cancer cell lines, including those from the lung, breast, and bladder. Its antiproliferative and pro-apoptotic effects are well documented, and some studies suggest enhanced efficacy when curcumin is used in combination with chemotherapeutic agents [16]. However, its poor bioavailability remains a challenge, which has prompted the development of alternative formulations such as nanoparticle encapsulation, liposomes, micelles, and phospholipid complexes [17].

In addition, homeopathy has been explored as an adjunct in cancer care to improve quality of life, and some ultra-diluted natural compounds have shown promising cytotoxic effects in vitro, although their mechanisms of action remain unclear [18,19].

Despite growing interest in curcumin, its use in canine bladder cancer has not yet been investigated. Likewise, reports on curcuminoids in other canine tumor types remain scarce. Therefore, the present study aimed to evaluate the in vitro effects of curcumin, D6 turmeric, and mitoxantrone hydrochloride on cell viability in canine and human urothelial carcinoma cell lines.

## 2. Materials and Methods

### 2.1. Experimental Design

This study investigated the antitumor effects of curcumin, D6 turmeric extract, and mitoxantrone hydrochloride—administered individually or in combination—on canine (SH, AXC and AXA) and human (T24) urothelial carcinoma cell lines. All cell lines were maintained under standard two-dimensional (2D) culture conditions. Treatments were applied individually and in combination. Cell viability was assessed using the MTT assay, while cell migration was evaluated via the Transwell assay. Apoptotic and necrotic cell populations were quantified by flow cytometry using Annexin V and propidium iodide staining. Additionally, total RNA was extracted for gene expression analysis via quantitative real-time PCR (qPCR), focusing on markers associated with epithelial–mesenchymal transition (EMT) and apoptosis.

### 2.2. Cell Culture

The canine high-grade urothelial carcinoma cell lines K9TCC-PU-AXA, K9TCC-PU-AXC, and K9TCC-PU-SH (abbreviated AXA, AXC, and SH, respectively) were kindly provided by Dr. Deborah Knapp (Purdue University, USA). These lines were derived from female dogs with grade I/II invasive UC. Notably, AXC and AXA were isolated from the same tumor via differential trypsinization [20]. The human T24 bladder carcinoma cell line (ATCC HTB-4™), donated by Dr. Fernanda Bueno Morrone (Pontifical Catholic University of Rio Grande do Sul, Brazil), originated from a grade III invasive tumor from an 81-year-old female patient [21]. All cell lines were confirmed to be free of mycoplasma contamination via PCR.

Cells were cultured in 25 cm^2^ and 75 cm^2^ flasks at 37 °C in a humidified incubator with 5% CO_2_. They were maintained in Ham’s F12 medium supplemented with 10–20% heat-inactivated fetal bovine serum (FBS), 1% gentamicin, and 0.5% antibiotic–antimycotic solution. Subculturing and cryopreservation were performed according to each line’s growth characteristics.

### 2.3. Curcumin, D6 Turmeric, and Mitoxantrone Hydrochloride Preparations

Curcumin (Sigma-Aldrich, St. Louis, MO, USA, C1386) was dissolved in dimethyl sulfoxide (DMSO) to prepare a 25 mg/mL stock solution. D6 turmeric, a homeopathic preparation of Curcuma longa at the sixth centesimal Hahnemannian dilution (6CH), was provided by InjectCenter© (Ribeirão Preto, Brazil) and diluted in physiological saline. Mitoxantrone hydrochloride (Evomixan^®^, Farmarin, Guarulhos, Brazil) was used in its commercial injectable form (20 mg/10 mL) and diluted in sterile water for the assays.

### 2.4. MTT Cell Viability Assay

Cell viability was assessed using the MTT assay, which quantifies mitochondrial metabolic activity. Cells (1 × 10^4^/well) were seeded in 96-well plates and treated with curcumin (12.000–18.000 ng/mL), D6 turmeric (5–20 µL/mL), or mitoxantrone hydrochloride (5–40 µM) for 72 h (curcumin and D6 turmeric) or 24 h (mitoxantrone) at 37 °C in 5% CO_2_. After treatment, MTT reagent (MTT, M2003, Sigma, Louis, MO) was added (0.5 mg/mL) and incubated for 4 h. Formazan crystals were dissolved in 200 µL DMSO per well. Absorbance was measured at 570 nm using a microplate reader (Biochrom Asys Expert Plus, Harvard Bioscience, Holliston, MA, USA) [22]. Vehicle controls matched the highest concentration of solvent used. IC_50_ values were calculated using non-linear regression with GraphPad Prism 8.0 (GraphPad Software, San Diego, CA, USA). All experiments were performed in triplicate.

### 2.5. Annexin V/Propidium Iodide Assay

Apoptosis and necrosis were evaluated using the Annexin V/Propidium Iodide (PI) apoptosis detection kit (Sigma-Aldrich, St. Louis, MO, USA), following the manufacturer’s instructions. Canine cells were treated with curcumin or mitoxantrone hydrochloride at their respective IC_50_ concentrations for 72 h and 24 h, respectively. Human T24 cells were treated only with curcumin. After treatment, the cells were trypsinized, centrifuged, and washed with PBS. Pellets were resuspended in 300 µL of binding buffer, followed by the addition of 5 µL Annexin V-FITC, 10 µL PI, and 5 µL Hoechst dye. Samples were incubated for 10 min in the dark at room temperature. The cells were analyzed by flow cytometry using an LSR Fortessa cytometer (BD Biosciences, Franklin Lakes, NJ, USA), and the data were processed with FACSDiva v6.2 software. Cell populations were classified as viable (Annexin V^−^/PI^−^), early apoptotic (Annexin V^+^/PI^−^), late apoptotic (Annexin V^+^/PI^+^), or necrotic (Annexin V^−^/PI^+^).

### 2.6. Quantitative Polymerase Chain Reaction (qPCR)

Cells (5 × 10^5^/well) were seeded in 6-well plates and treated for 24 h (mitoxantrone) or 72 h (curcumin and D6 turmeric) using IC_50_ concentrations. Total RNA was extracted using TRIzol^®^ reagent (Invitrogen, Waltham, MA, USA) and stored at −80 °C. RNA quantity and purity were assessed using a NanoDrop ND-1000 spectrophotometer (Thermo Fisher Scientific, Waltham, MA, USA). Reverse transcription was performed using a commercial kit (Applied Biosystems, Waltham, MA, USA), and cDNA was diluted 1:10 prior to qPCR. Primer sequences were designed using Primer Express 3.0 (Applied Biosystems). Amplification was performed using PowerUp SYBR Green Master Mix (Applied Biosystems™, Thermo Fisher Scientific, Waltham, MA, USA). Gene expression was calculated using the 2^−ΔΔCt^ method. Target genes included *β-catenin*, *β1-integrin*, *CDH1*, *MMP-2*, *MMP-9*, and *TIMP-2*. Species-specific primers for canine and human gene expression analysis. Canine primer sequences are shown in Table 1.

### 2.7. In Vitro Migration Assay

Cell migration was assessed using Transwell chambers (6.5 mm diameter, 8 µm pore size; Corning, Glendale, AZ, USA) in 24-well plates. SH and T24 cells (5 × 10^4^ cells/well) were seeded into the upper chamber in serum-free medium containing vehicle or IC_50_ concentrations of curcumin, D6 turmeric, or mitoxantrone hydrochloride. The lower chamber contained 800 µL of DMEM with 10% FBS as a chemoattractant. After 24 h of incubation at 37 °C, non-migrating cells were removed with a cotton swab. Migrated cells on the lower surface were fixed in 100% methanol for 8 min and stained with Giemsa (Merck KGaA, Darmstadt, Germany) for 15 min. Cell migration was quantified by counting cells in four random fields per membrane (200× magnification). Each treatment was performed in quadruplicate.

### 2.8. Statistical Analysis

Data were expressed as mean ± standard deviation (SD) from at least three independent experiments. Statistical significance was determined using the unpaired Student’s *t*-test, with *p* < 0.05 considered significant. GraphPad Prism 8.0 (GraphPad Software, San Diego, CA, USA) was used for all analyses. Transwell image quantification was performed using ImageJ software (version 1.53k; National Institutes of Health, Bethesda, MD, USA).

## 3. Results

### 3.1. MTT Cell Viability Assay and Determination of IC_50_ After Treatments

The cytotoxic effects of curcumin, D6 turmeric, and mitoxantrone hydrochloride were evaluated in canine (AXA, AXC, SH) and human (T24) urothelial carcinoma cell lines using the MTT assay.

**Figure 1:**
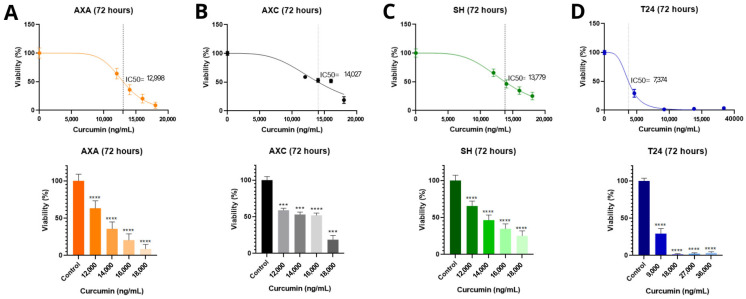
Effect of curcumin on cell proliferation and mean of IC_50_ values of K9TCC-PU AXA (**A**), AXC (**B**), and SH (**C**) canine and human T24 (**D**) cell lines after 72 h of incubation. *** *p* < 0.001 and **** *p* < 0.0001. Vertical intermittent lines show IC_50_ dose and colored dots represent the drug dose (ng/mL).

As shown in Figure 1, curcumin exhibited a dose-dependent cytotoxic effect in all four cell lines after 72 h. The IC_50_ values for the canine lines were relatively similar—12.996 ng/mL (AXA), 14.027 ng/mL (AXC), and 13.779 ng/mL (SH)—indicating a consistent response. In contrast, the human T24 cell line was more sensitive to curcumin, with a lower IC_50_ value of 7.374 ng/mL.

**Figure 2:**
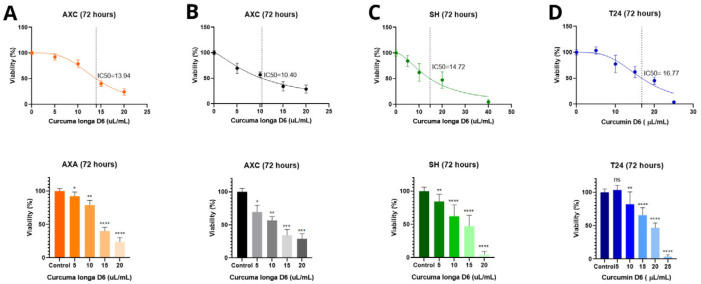
Effect of D6 turmeric on cell proliferation and mean IC_50_ values of K9TCC-PU AXA (**A**), AXC (**B**), and SH (**C**) canine and human T24 (**D**) cell lines after 72 h of incubation. * *p* < 0.05, ** *p* < 0.01, *** *p* < 0.001, **** *p* < 0.0001, and ns = not significant. Vertical intermittent lines show IC_50_ dose and colored dots represent the drug dose (µL/mL).

D6 turmeric produced a comparable inhibitory effect in the canine cell lines. The IC_50_ values were 13.94 µL/mL (AXA), 10.40 µL/mL (AXC), and 14.72 µL/mL (SH). However, in the T24 line, D6 turmeric showed a higher IC_50_ value of 16.77 µL/mL, with no significant cytotoxicity observed at 5 µL/mL (Figure 2).

**Figure 3:**
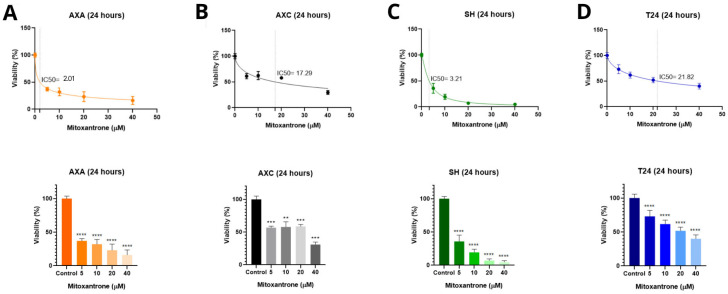
Effect of mitoxantrone hydrochloride on cell proliferation and mean IC_50_ values of K9TCC-PU AXA (**A**), AXC (**B**), and SH (**C**) canine and human T24 (**D**) cell lines after 24 h of incubation. ** *p* < 0.01, *** *p* < 0.001, and **** *p* < 0.001. Vertical intermittent lines show IC_50_ dose and colored dots represent the drug dose (µM).

Mitoxantrone hydrochloride treatment for 24 h resulted in the lowest IC_50_ values in AXA (2.01 µM) and SH (3.21 µM), indicating high sensitivity. The AXC and T24 cells showed greater resistance, with IC_50_ values of 17.29 µM and 21.82 µM, respectively. The AXC line maintained viability across the lower concentrations (5, 10, and 20 µM), while the T24 line demonstrated a dose-dependent response (Figure 3).

### 3.2. Cell Death Analysis by Annexin V/PI Staining

**Figure 4:**
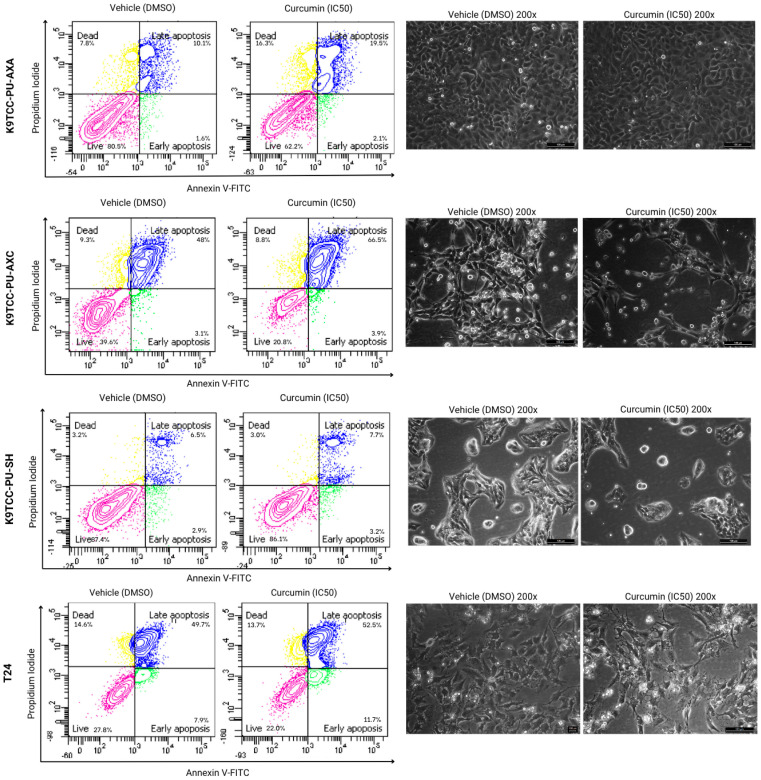
Annexin V-FITC/PI after 72 h of incubation with curcumin in canine AXA, AXC, and SH, and human T24 tumor cells. The upper left quadrant (yellow) represents the group of necrotic cells, and the one on the upper right (blue), the group of cells in late apoptosis. The cells in the lower right quadrant (green) represent the group of cells in early apoptosis and the lower left quadrant (pink), the viable cells.

Flow cytometry analysis using Annexin V/PI staining revealed that curcumin significantly increased the proportion of late apoptotic and necrotic cells in the AXA and SH cell lines at the IC_50_ concentration (13.000 ng/mL) after 72 h. In the AXC cells, curcumin notably increased late apoptosis (from 48% to 66.5%) at 14.000 ng/mL. No significant effect on necrosis was observed in the SH cells. In the T24 cells, curcumin enhanced both early and late apoptotic populations (Figure 4).

**Figure 5:**
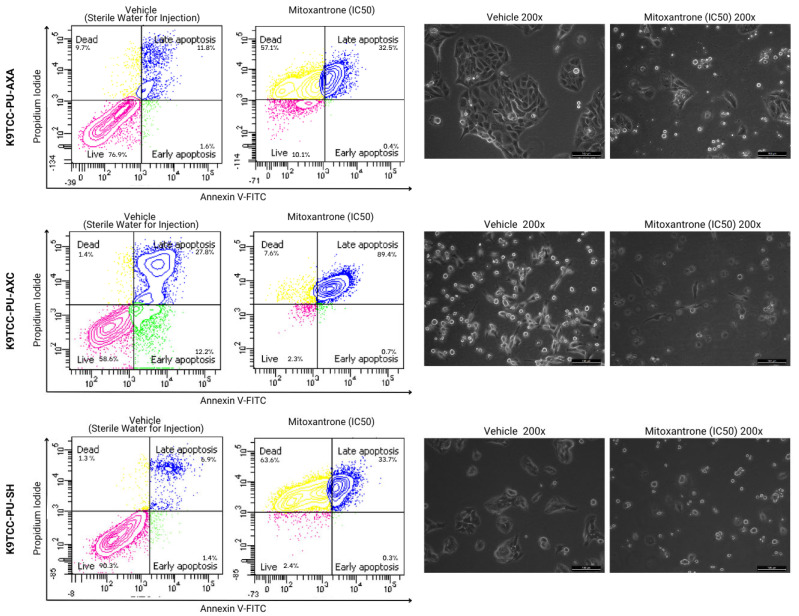
Annexin V-FITC/PI after 24 h of incubation with mitoxantrone hydrochloride in canine AXA, AXC, and SH cells. The upper left quadrant (yellow) represents the group of necrotic cells, and the one on the upper right (blue), the group of cells in late apoptosis. The cells in the lower right quadrant (green) represent the group of cells in early apoptosis and the lower left quadrant (pink), the viable cells.

Mitoxantrone hydrochloride also induced late apoptosis in all canine cell lines. Additionally, the AXA and SH cells showed an increase in necrotic populations. The T24 cells were not evaluated in this assay due to an insufficient number of cells for analysis (Figure 5).

### 3.3. Effects of Treatment on Cell Migration

**Figure 6:**
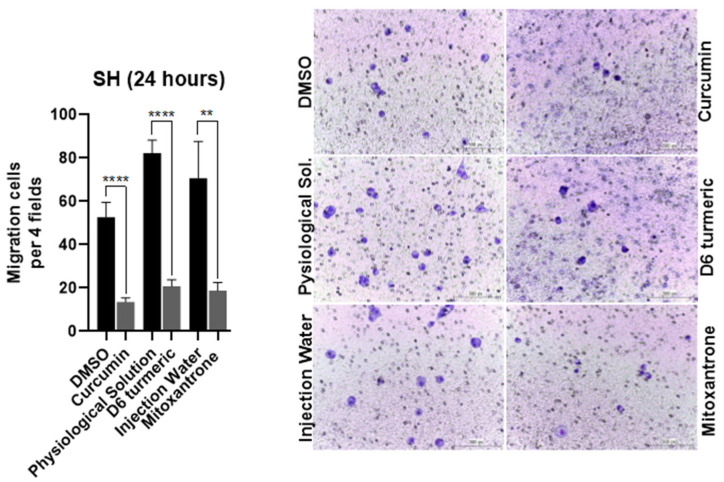
The cell migration assay of canine urothelial bladder carcinoma cells (SH) treated with curcumin, D6 turmeric, and mitoxantrone hydrochloride and their respective vehicles. The bar graphs represent the mean account of cells in four regions of each well randomly (**left**). The representative microscopy images of the migration assay show SH cells treated with compounds or vehicles for 24 h, Giemsa, obj 200× (**right**). ** *p* < 0.01; **** *p* < 0.0001.

**Figure 7:**
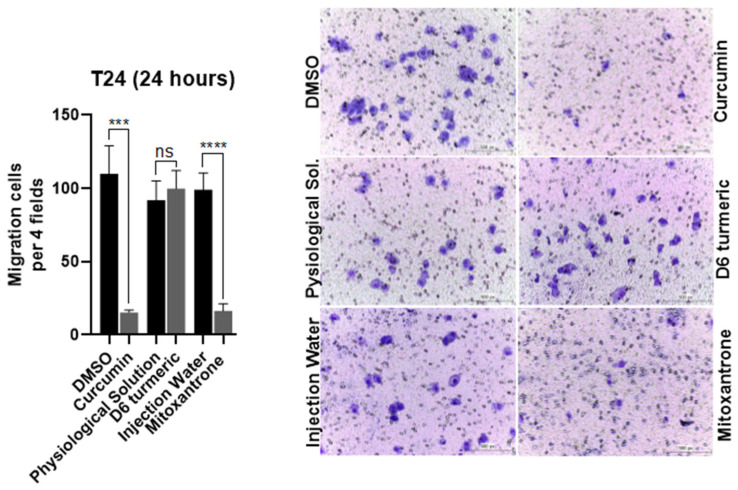
The cell migration assay of human urothelial bladder carcinoma cells (T24) treated with curcumin, D6 turmeric, and mitoxantrone hydrochloride and their respective vehicles. The bar graphs represent the mean account of cells in four regions of each well randomly (**left**). The representative microscopy images of the migration assay show T24 cells treated with compounds or vehicles for 24 h, Giemsa, obj 200× (**right**). *** *p* < 0.001; **** *p* < 0.0001, and ns = not significant.

Transwell migration assays showed that curcumin, D6 turmeric, and mitoxantrone hydrochloride (IC_50_) significantly inhibited SH cell migration after 24 h—curcumin (75.8%), D6 turmeric (74.8%), and mitoxantrone (73.6%) (Figure 6). In the human T24 line, curcumin and mitoxantrone inhibited migration by 86.14% and 83.59%, respectively. D6 turmeric did not significantly affect T24 cell migration (Figure 7).

### 3.4. Quantitative Polymerase Chain Reaction

**Figure 8:**
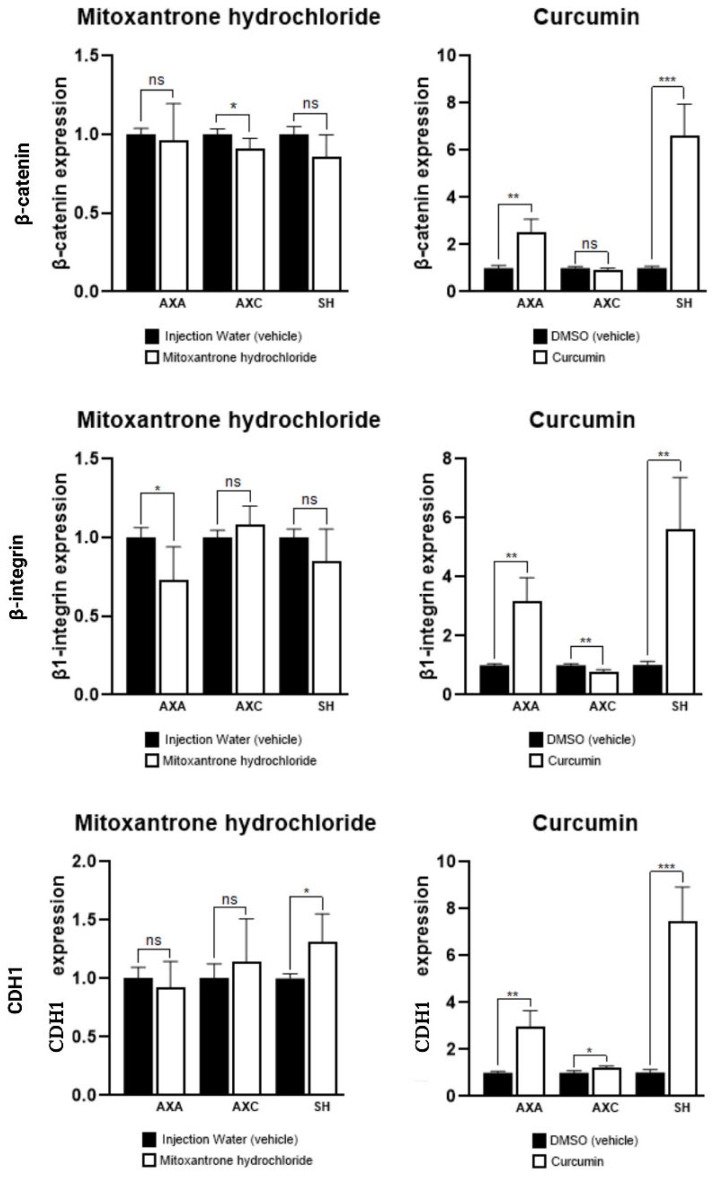
Gene expression of *β-catenin*, *β1-integrin*, and *CDH1* in canine urothelial carcinoma cells treated with mitoxantrone hydrochloride or curcumin, in comparison with respective control cells (vehicle). * *p* < 0.001, ** *p* < 0.001, *** *p* < 0.0001, and ns = not significant.

Gene expression analysis revealed that curcumin significantly upregulated EMT-related genes *β-catenin*, *β1-integrin*, and *CDH1* in AXA and SH cells. In contrast, mitoxantrone treatment led to a downregulation of *β-catenin* in AXC and *β1-integrin* in AXA cells (Figure 8).

**Figure 9:**
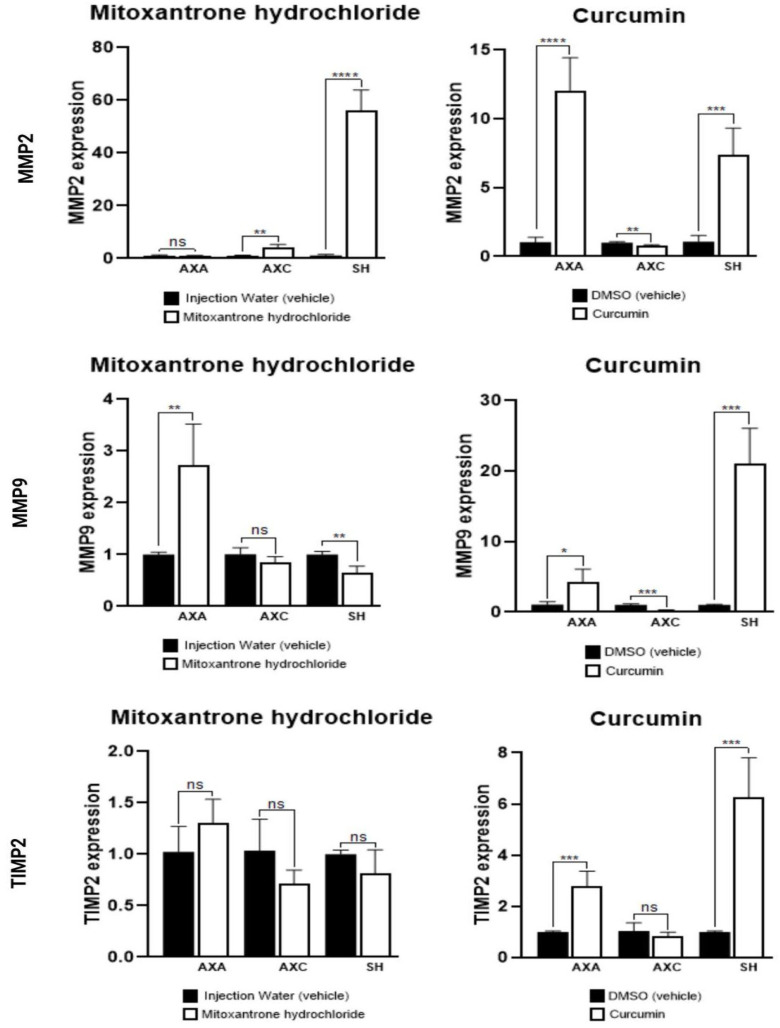
Gene expression of *MMP-2*, *MMP-9*, and *TIMP-2* in canine urothelial carcinoma cells treated with mitoxantrone hydrochloride or curcumin, in comparison with respective control cells (vehicle). * *p* < 0.001, ** *p* < 0.001, *** *p* < 0.0001, **** *p* < 0.0001, and ns = not significant.

Curcumin treatment increased MMP-2 and MMP-9 expression in all canine cell lines and upregulated *TIMP-2* in AXA and SH. Mitoxantrone elevated *MMP-2* in AXC and SH, and MMP-9 in AXA and SH (Figure 9).

**Figure 10:**
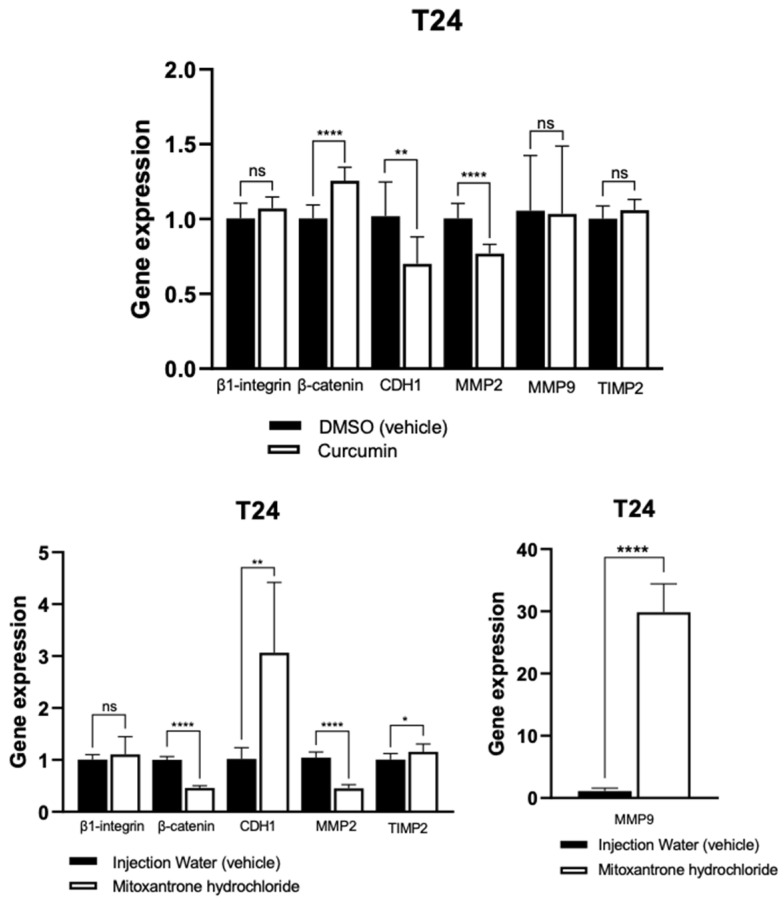
Gene expression of *β-catenin*, *β1-integrin*, *CDH1*, *MMP-2*, *MMP-9*, and *TIMP-2* in human urothelial carcinoma cells treated with curcumin and mitoxantrone hydrochloride, in comparison with respective control cells (vehicle). * *p* < 0.001, ** *p* < 0.0001, **** *p* < 0.0001, and ns = not significant.

In the T24 human cell line, curcumin upregulated β-catenin and downregulated MMP-2. Mitoxantrone increased CDH1 and MMP-9 expression but decreased β-catenin and MMP-2 levels. TIMP-2 expression was not significantly altered (Figure 10).

## 4. Discussion

Urothelial carcinoma is the most common neoplasm in the canine urinary tract, frequently occurring in the vesical trigone with the involvement of the prostatic urethra, which complicates surgical intervention [23]. Routine chemotherapy protocols improve clinical symptoms; however, response rates and median survival time remain short, ranging from 4 to 10 months [24]. Chemotherapy treatment, whether combined with non-steroidal anti-inflammatory drugs (NSAIDs), has shown varying degrees of success [25].

The search for natural treatments as a response to cancer is receiving a lot of attention. Many commonly used chemotherapy compounds are toxic to both tumor cells and normal cells, leading to numerous side effects [26]. New drugs, particularly natural compounds like curcumin, are being looked into, as they can be combined with the chosen chemotherapeutic treatments to help relieve their side effects [27]. Curcumin has shown effectiveness in treating chronic diseases, including various cancers in both humans and dogs [28]. Moreover, it exhibits selectivity in killing tumor cells while preserving normal cells, impacting multiple signaling pathways, and reducing the likelihood of tumor cell resistance developing [26].

In this study, curcumin was observed as effectively reducing cell viability in three canine UC cell lines, with concentrations as low as 12.996 ng/mL (AXA) and as high as 14.027 ng/mL (AXC) after 72 h of exposure. These results are in accordance with previous experiments that have also demonstrated in vitro curcumin’s cytotoxic effect in canine and human osteosarcoma cell lines. The researchers observed antitumor activity, reduced migration, and inhibited cell viability when using curcumin encapsulated in a liposomal form [29,30]. Curcumin’s antiproliferative effects on human T24 UC cells did not generate a dose-effect curve. Additionally, our results showed an IC_50_ value that was lower compared to the canine cells, 7.374 ng/mL. In contrast, Shi et al. (2017) [31] demonstrated reduced T24 cell viability in a time- and dose-dependent manner, with an IC_50_ value of 10 µM/L (10.000 ng/mL) curcumin in vitro.

Mitoxantrone hydrochloride is established as the drug of choice in the treatment of various canine neoplasms, including UC, with lower cardiotoxicity effects than doxorubicin and presenting better results when combined with Piroxicam [32,33]. Canine cell lines were most responsive to mitoxantrone hydrochloride treatment compared to the human cell line, with IC_50_ values of 2.01 µM (AXA), 3.21 µM (SH), 17.29 µM (AXC), and 21.82 µM (T24). Previous results have been published with higher IC_50_ values for the same canine UC cell lines treated with mitoxantrone [25].

Cell migration is one of the most important features that contribute to metastasis. Curcumin and mitoxantrone were able to reduce cell migration in canine UC in SH and human T24 cells. D6 turmeric did not show a reduction in cell migration in the T24 UC cell line, but had an effect on the canine cell lines.

Canine bladder UC is a highly invasive tumor, with 16% of dogs already presenting invasion and metastasis at the time of diagnosis. Lymphatic invasion has been related to shorter overall survival time in dogs with bladder UC, than in animals without lymphatic invasion [34]. Our results, associated with previously published results with curcumin in the T24 cell line [31], encourage further in vitro and in vivo studies with curcumin for both humans and dogs.

The natural compound curcumin showed cell death results similar to mitoxantrone for the canine and human BUC cell lines, with curcumin increasing early and late apoptosis. A previous in vitro study demonstrated that mitoxantrone induces apoptosis in cancer cells by inhibiting RNA and DNA synthesis, binding to the topoisomerase II enzyme, and involving various anticancer mechanisms such as autophagy, paraptosis, radiosensitization, and aberrant cell metabolism [35]. In vitro studies on this chemotherapeutic are limited and require further investigation, particularly regarding the commercial presentation studied in our research.

Curcumin significantly increases the number of late apoptotic and necrotic canine UC cells, particularly in the AXC lineage. Soares et al. [36] conducted an in vitro study using canine osteosarcoma cells and observed the apoptotic effects mediated by turmeric through the JNK and cAMP/AMPK pathways, which are involved in the production of the p38 and p53 proteins. Our study also suggests that curcumin induces both early and late apoptosis in T24 cells, consistent with the findings reported earlier by Shi et al. [31] after 24 h of treatment. Genes related to cell death and apoptosis in canine UC cells treated with curcumin require exploration in future research.

EMT is a relevant mechanism of tumor progression in neoplasms of epithelial origin, related to invasion and metastasis. Our work demonstrated the upregulation of *β-catenin*, *β-integrin*, and *CDH1* in the AXA and SH canine UC cell lines after curcumin treatment. E-cadherin is an important epithelial cell adhesion protein, codified by the *CDH1* gene. It is interesting to observe the upregulation of this gene after curcumin treatment in both human and canine UC cell lines, since it can demonstrate an increase in E-cadherin expression, less epithelial cell detachment from one another, and consequently, a lower probability of invasion. Bahrami et al. [37] reported that curcumin had an inhibitory effect on the EMT process in distinct types of human neoplasms, including oral, colorectal, thyroid, and lung cancer.

Previous research demonstrated the upregulation of *CDH1* in human colorectal cancer cells and the T24 human lineage, and, to the authors knowledge, this is the first time it is described in canine UC cell lines [38]. An interesting finding in the SH canine UC cell line showed the highest CDH1 expression after curcumin treatment, and the SH cell line is from a xenograft model of a lymph node metastasis, representing an aggressive tumor.

The observed changes in gene expression suggest an intricate interplay between adhesion molecules, EMT markers, and matrix remodeling enzymes that may collectively influence the metastatic potential of urothelial carcinoma cells. For instance, the upregulation of *CDH1* in canine and human cell lines after curcumin treatment may reflect a partial reversion of EMT, potentially reinforcing epithelial characteristics and reducing cellular detachment [39]. This effect is particularly relevant in the SH canine cell line, which originated from a metastatic lymph node and showed the highest *CDH1* expression post-treatment.

In normal cells, E-cadherin regulates *β-catenin* translocation to the cell nucleus and proliferation stimuli. The association and high affinity of the *CDH1*/*β-catenin* complex are important in maintaining epithelial morphology and can be disrupted during the oncogenesis process [40]. On the other hand, high levels of nuclear *β-catenin* induce cancer proliferation, survival, and T-cell suppression [41]. Some human cancers, including breast, colorectal, prostate, and ovarian, have shown high *β-catenin* expression levels downregulated by curcumin [42]. Further studies should be conducted to understand the role of curcumin treatment in the upregulation of *β-catenin* and its role as an anti- or pro-neoplastic agent.

Concurrently, the increased expression of *β-catenin* in the same treatment conditions raises important considerations. While *β-catenin* is associated with adherens junctions through its interaction with E-cadherin, it also plays a dual role in cancer, as its nuclear translocation is linked to oncogenic signaling [43]. Therefore, the co-upregulation of *CDH1* and *β-catenin* could reflect the stabilization of cell–cell adhesion [44].

We also observed the upregulated expression of *β1-integrin* in the AXA and SH canine cells after curcumin treatment. High *β1-integrin* regulation in cancer cells promotes the proliferation, survival, migration, and invasion capacity derived from adhesion between cancer cells and the extracellular matrix (ECM) [45]. However, in opposition to this, the downregulation of AXA *β1-integrin* reveals the potential use of mitoxantrone hydrochloride for anti-metastasis and anti-invasion approaches.

The modulation of *β1-integrin*, an important mediator of tumor cell interaction with the extracellular matrix, adds another layer of complexity. Its upregulation by curcumin in AXA and SH cells may indicate a context-dependent response, as *β1-integrin* is known to support both cell survival and migration. Conversely, its downregulation by mitoxantrone in AXA cells suggests potential anti-invasive effects.

*MMP2* and *MMP9* are invasion-associated gelatinases responsible for extracellular matrix degradation and subsequent tissue invasion, and they have been described with elevated expression in canine neoplasms [46]. In this study, curcumin and mitoxantrone hydrochloride were effective at inhibiting in vitro migration, evaluated by the Transwell test, but *MMP9* and *MMP2* gene expression might not be correlated to that, since each cell line showed a different expression (the up- or downregulation of *MMP9* and *MMP2*). Also, there was no correlation with the increase in metalloproteinase gens and their regulator, *TIMP-2*.

Furthermore, *MMP-2* and *MMP-9*, key proteases involved in ECM degradation and invasion, were upregulated in most canine cell lines following curcumin treatment [47]. Interestingly, this increase was accompanied by an upregulation of *TIMP-2* in some cases (e.g., AXA and SH), which may represent a feedback mechanism to counterbalance the proteolytic activity of MMPs [48]. However, this molecular signature did not fully align with the results from the migration assay, where curcumin significantly reduced migration in both canine and human cells. This discrepancy underscores the complex regulation of invasion, which likely depends not only on MMP/TIMP expression but also on other post-translational modifications and cellular context [49].

The co-expression of *MMP-2* and *TIMP-2* may indicate a dynamic regulatory response, as the upregulation of both *MMP-2* and its inhibitor *TIMP-2* could signify a feedback mechanism intended to maintain equilibrium in matrix degradation, even in the absence of a genuine extracellular matrix substrate. Furthermore, *TIMP-2* is not solely an inhibitor; it also plays a crucial role in the activation of pro-MMP-2 through the formation of complexes with MT1-MMP on the cell surface [48,49].

Curcumin treatment in the T24 cell line showed better results for EMT gene expression. *β-catenin* was upregulated with curcumin treatment and *MMP2* was downregulated compared to the control group. Although there was no difference in *MMP9* expression with curcumin treatment, mitoxantrone upregulated this gene, an effect that can facilitate cellular invasion. The process of EMT, invasion, and metastasis involves many signaling pathways and interrelationships between different molecules [50]. For this reason, more research needs to be developed to elucidate the mechanisms of curcumin and mitoxantrone hydrochloride in this process.

Taken together, these findings emphasize that EMT, adhesion, and invasion-related genes operate in interconnected pathways, rather than in isolation. The collective behavior of these markers suggests that curcumin may modulate multiple signaling cascades with potential antimetastatic outcomes [51]. However, these hypotheses must be validated through functional assays and in vivo studies, including analyses of protein expression, localization, and pathway activation status.

It is important to acknowledge some limitations of the present study. Although the tested compounds may potentially affect non-tumor cells and organs, our experimental design focused on evaluating their antitumoral activity in urothelial carcinoma cells. This approach aimed to investigate whether the combination of a phytochemical agent with a chemotherapeutic drug could enhance cytotoxicity in tumor cells, potentially allowing dose reduction and improved safety.

Furthermore, we recognize that while in vitro studies provide critical initial evidence, they do not fully replicate the complexity of in vivo systems. Therefore, additional studies using animal models are planned to further investigate the efficacy, pharmacokinetics, and systemic toxicity of these compounds. These efforts will be essential to determine the clinical applicability of curcumin and D6 turmeric in veterinary oncology.

Our study is a pioneer in the in vitro use of turmeric D6 in canine and human UC, as well as curcumin in canine UC, demonstrating an antiproliferative effect and a decrease in cell migration. There is a preoccupation with its use in vivo, due to its low bioavailability in tissues after administration [48]. However, with these promising results, more research could be developed to better understand the mechanism of action, as well as the association with products to increase and facilitate its distribution and viability.

## 5. Conclusions

D6 turmeric, curcumin, and mitoxantrone hydrochloride were effective in reducing the viability of canine and human UC cell lines in culture. Furthermore, the study revealed a reduction in migratory cell activity when employing D6 turmeric in both canine and human cell lines. These are the first results of the effect of curcumin and D6 turmeric on canine and human UC. However, further research is needed to evaluate the mechanism of action and the form of administration of the treatment, local or oral.

Treatment response was not similar between canine UC cell lines in terms of the expression of invasion and EMT genes, which may suggest distinct gene signatures for each neoplasm and encourage personalized treatment. The association of chemotherapeutic agents with natural compounds, such as curcumin, offers new possibilities for the in vivo treatment of humans and dogs with bladder urothelial carcinoma.

## Figures and Tables

**Table 1:** Canine primer sequences.

Targets	Primers Sequences (5′–3′)
*β-catenin* canine	Forward: 5′-GATACCCAGCGCCGTACGT-3′ Reverse: 5′-GACCCCCTCCACAAATTGC-3′
*β1-integrin* canine	Forward: 5′-AGCCATTTGCAAGGTTTTATCC-3′ Reverse: 5′-GGATTCAGGGTTTCTCAGATGTTAA-3′
*CDH1* canine	Forward: 5′-CAGGCCTCCGTTTCTGGAA-3′ Reverse: 5′-GGAGAGGAGTTGGGAAATGTG-3′
*MMP-2* canine	Forward: 5′-TGAGCTATGGACCTTGGGAGAA-3′ Reverse: 5′-CCATCGGCGTTCCCATAC-3′
*MMP-9* canine	Forward: 5′-GGACGATGCCTGCAACGT-3′ Reverse: 5′-CAAATACAGCTGGTTCCCAATCT-3′
*TIMP-2* canine	Forward: 5′-GGGCCAAAGCGGTCAGT-3′ Reverse: 5′-TAGGGTTGCCATAAATGTCGTTT-3′
*β-catenin* human	Forward: 5′-GATACCCAGCGCCGTACGT-3′ Reverse: 5′-GACCCCCTCCACAAATTGC-3′
*β1-integrin* human	Forward: 5′-AGCCATTTGCAAGGTTTTATCC-3′ Reverse: 5′-GGATTCAGGGTTTCTCAGATGTTAA-3′
*CDH1* human	Forward: 5′-CAGGCCTCCGTTTCTGGAA-3′ Reverse: 5′-AGGAGAGGAGTTGGGAAATGTG-3′
*MMP-2* human	Forward: 5′-TGAGCTATGGACCTTGGGAGAA-3′ Reverse: 5′-CCATCGGCGTTCCCATAC-3′
*MMP-9* human	Forward: 5′-GGACGATGCCTGCAACGT-3′ Reverse: 5′-CAAATACAGCTGGTTCCCAATCT-3′
*TIMP-2* human	Forward: 5′-GGGCCAAAGCGGTTCAGT-3′ Reverse: 5′-TAGGGTTGCCATAAATGTCGTTT-3′

## Data Availability

The original contributions presented in this study are included in the article. Further inquiries can be directed to the corresponding author.
